# Forager bees (*Apis mellifera*) highly express immune and detoxification genes in tissues associated with nectar processing

**DOI:** 10.1038/srep16224

**Published:** 2015-11-09

**Authors:** Rachel L. Vannette, Abbas Mohamed, Brian R. Johnson

**Affiliations:** 1 Department of Biology, Stanford University, 488 Herrin Labs, Stanford, 94043, United States; 2 Department of Entomology and Nematology, University of California, 043 Briggs Hall Davis, CA 95616, United States.

## Abstract

Pollinators, including honey bees, routinely encounter potentially harmful microorganisms and phytochemicals during foraging. However, the mechanisms by which honey bees manage these potential threats are poorly understood. In this study, we examine the expression of antimicrobial, immune and detoxification genes in *Apis mellifera* and compare between forager and nurse bees using tissue-specific RNA-seq and qPCR. Our analysis revealed extensive tissue-specific expression of antimicrobial, immune signaling, and detoxification genes. Variation in gene expression between worker stages was pronounced in the mandibular and hypopharyngeal gland (HPG), where foragers were enriched in transcripts that encode antimicrobial peptides (AMPs) and immune response. Additionally, forager HPGs and mandibular glands were enriched in transcripts encoding detoxification enzymes, including some associated with xenobiotic metabolism. Using qPCR on an independent dataset, we verified differential expression of three AMP and three P450 genes between foragers and nurses. High expression of AMP genes in nectar-processing tissues suggests that these peptides may contribute to antimicrobial properties of honey or to honey bee defense against environmentally-acquired microorganisms. Together, these results suggest that worker role and tissue-specific expression of AMPs, and immune and detoxification enzymes may contribute to defense against microorganisms and xenobiotic compounds acquired while foraging.

Pollinator populations are threatened by numerous environmental and anthropogenic factors^1^,^2^. Microorganisms and xenobiotic compounds encountered while foraging may contribute to pollinator decline^2^,^3^,^4^, but recent work suggests that pollinators can respond dynamically to such challenges through changing expression of immune and detoxification genes^5^,^6^. However, inadequate knowledge of gene expression patterns among pollinators that vary in exposure to environmentally acquired threats limits our ability to interpret these experimental results.

The honey bee (*Apis mellifera)*, a polylectic social pollinator, routinely encounters potentially hazardous microorganisms and chemical compounds while foraging. Diverse communities of yeasts and bacteria inhabit flowers, and often attain high abundance in floral nectar^7^,^8^,^9^,^10^,^11^. The yeasts and acetic acid bacteria within nectar ferment nectar sugars and produce ethanol and organic acids^12^,^13^,^14^, which may interfere with honey conversion and storage processes^15^,^16^. As a pollinator that relies on stored floral resources, the western honey bee *Apis mellifera* may be vulnerable to microbial degradation of stored nectar resources, particularly during nectar flows and before low water activity prevents microbial degradation^17^,^18^. Additionally, floral nectar frequently contains phytochemicals with potential toxicity against a range of arthropods^19^,^20^. However, current evidence indicates that honey bees and other social pollinators are relatively tolerant to ecologically relevant concentrations of nectar secondary compounds^21^,^22^. Furthermore, pollinators can also maintain a relatively stable individual and hive microbiome^23^,^24^ and examples of honey spoilage and chemical lethality to bees themselves from naturally-occurring compounds are rare^21^, begging the question how honey bees can effectively cope with such microbial and chemical challenges^25^.

Honey bees employ a variety of strategies to protect individuals and the colony from pathogens, parasites, and exposure to xenobiotics^23^,^26^. Previous work has demonstrated that honey bees alter their behavior and gene expression after exposure to pathogens or xenobiotics associated with colony collapse disorder^27^,^28^. However, most recent work has focused on transcriptional responses in the bee midgut or haemolymph^5^. This approach has identified key genes that mediate the individual bee’s response to pathogen challenge or xenobiotic exposure. However, in many cases, microorganisms and compounds may not be immediately consumed and are instead stored in honey or beebread, or passed on to in-hive workers for consumption. Food gathering and processing is performed by forager bees, which produce sugar conversion and preservation enzymes in the hypopharyngeal gland. Because of the changing roles of worker bees in the colony, and their differential exposure to environmental hazards^18^, including chemical compounds and microorganisms, workers may also vary in their investment in mechanisms to cope with these challenges. Previous work has shown that honey bee immune response can change through ontogeny^29^, but if worker castes vary systematically in the transcription of defensive or immune-related genes is not well understood.

Here, we use tissue-specific RNA-seq^30^ to analyze transcriptome-wide gene expression between nurses (young workers that care for brood) and foragers (older workers that forage for and process nectar). We compare gene expression in the hypopharyngeal gland (HPG) and mandibular glands, tissues associated with honey production, to the midgut and Malpighian tubules, tissues involved in metabolism and toxin excretion. We subsequently used qPCR to analyze a subset of differentially expressed genes identified using RNA-seq, on independent samples. Our analyses focus on the expression of genes involved in immunity, including those encoding antimicrobial peptides or proteins involved in immune signaling, and those involved in detoxification, including cytochrome P450 monooxygenases (Supplementary table S1 online).

## Results

**Figure 1:**
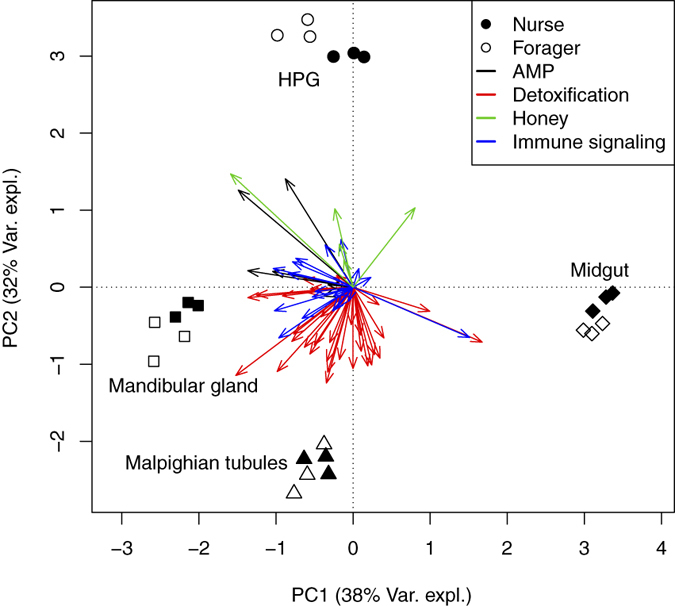
Distance biplot of principle components analysis (PCA) depicting distance between tissues in honey bee (*Apis mellifera*) based on difference in expression of genes involved in immune signaling, and the production of antimicrobial peptides, honey processing enzymes and detoxification genes. Points represent biological replicates within each tissue and caste, with closed points for nurse bees and open points for forager bees. The distance between points approximates difference in gene expression patterns among samples. Arrows represent different genes that were differentially expressed among tissues or between nurses and foragers. Arrow color corresponds to the functional class of genes, and distance between arrowheads approximates difference in their (log-transformed) expression among tissues. Arrowheads close to a particular tissue type are expressed at highest abundance in those samples. Gene labels are omitted for clarity.

Honey bee tissues varied substantially in the abundance of particular transcripts, and specifically, of those encoding products with antimicrobial, immune signaling and detoxification functions (Supplementary Figures S2 and S3, Table 1; Fig. 1; perMANOVA F_1,23_ = 11.63, P < 0.001). The HPG was enriched in transcripts for antimicrobial peptides (AMPs) and honey-producing enzymes, while each tissue type was enriched with transcripts from different immune signaling and detoxification genes (Table 1). The Malpighian tubules and the midgut were both enriched in detoxification genes, including cytochrome P450s monooxygenases (P450s), carboxyl/cholinesterases (CCEs) and glutathione S-transferases (GSTs) and many were highly expressed (Fig. 1, Table 1).

**Figure 2:**
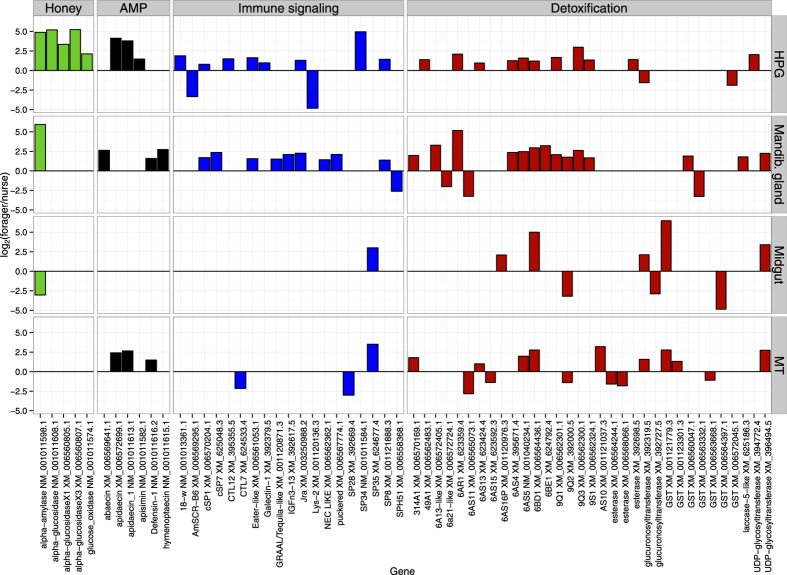
Log_2_ fold change in the expression of individual genes between honey bee foragers and nurses. Values above zero indicate greater relative transcript abundance in foragers compared to nurses. Genes included were differentially expressed at FDR < 0.05 in edgeR. The color of each gene indicates its putative function, following colors used in Fig. 1. See Methods for full details.

Within tissues, nurses and foragers differed in expression of genes with putative antimicrobial, signaling and detoxification functions, and other tissue-specific transcripts (Fig. 2). As expected, the HPG of foragers was enriched with honey processing genes, including *α-amylase* and *glucose oxidase* (Fig. 2). In contrast, the nurse HPG was highly enriched in transcripts for major royal jelly proteins used to produce brood food, typically considered the main function of the HPG in nurse bees^31^, as has previously been described^32^. Of chief importance, two antimicrobial peptides (*apisimin* and *defensin*) were highly coexpressed with nectar processing enzymes in HPG and mandibular glands of foragers, but not nurses (Figs 1 and 2).

Overall, foragers expressed genes encoding antimicrobial peptides (AMPs) to a greater extent than nurses across nearly all tissues (Fig. 2), although relative expression levels were much greater in tissues associated with nectar processing and social interactions. For example, in the forager HPG, *apisimin* was expressed at nearly ~1,150,000 RPKM, over 14 times greater than expression of the nectar-conversion enzyme glucose oxidase, and two variants of *apidaecin* were also highly expressed. Forager mandibular glands were also enriched in transcripts coding for antimicrobial peptides compared to nurses (Fig. 2), with *defensin-1* and *hymenoptaecin* expressed at high levels (4993 and 1299 RPKM respectively). Forager Malpighian tubules were also enriched in AMP-encoding transcripts including *apidaecin* and *defensin-1*, but expression levels were nearly 1000 times lower than in the HPG. In contrast, expression of antimicrobial transcripts in the midgut did not differ between nurses and foragers.

Transcripts associated with immune signaling pathways were also enriched in foragers compared to nurses (Fig. 2), but this difference was only observed in the HPG and mandibular glands (Fig. 2). Forager HPGs were enriched in transcripts encoding *Jra*, *Galectin*-1, *eater-like*, and *PPOAct*, and *CTL12*, as well as multiple serine protease genes, which encode recognition proteins and signaling molecules involved in the JNK pathway and phagocytosis. Similarly, forager mandibular glands were enriched transcripts coding for the immune-related genes including *puckered*, *NEC LIKE, Jra, IGFn-3-13, GRAAL (Tequila-like), PPOAct* and *eater-like and* four serine proteases. In the Malpighian tubules, nurses were enriched in a single immune signaling gene (*CTL7*) and one serine protease, and foragers in one serine protease. In contrast, expression of immunity-related genes in the midgut was similar between nurses and foragers, although foragers were enriched in one serine protease (Fig. 2).

Foragers and nurses also differed in their expression of many detoxification-related genes, particularly in the HPG and mandibular gland, and to a lesser extent in the Malpighian tubules and midgut (Fig. 2). Forager HPGs were enriched in many putative detoxification transcripts, including those encoding enzymes from the *CYP6AS* and *CYP9Q* subfamilies of P450s (Fig. 2). Forager mandibular glands were also highly enriched in transcripts encoding putative detoxification enzymes, including P450s, with representatives from the *CYP9Q*, *CYP6BD*, *CYP9S*, and *CYP6AS* subfamilies and glutathione-S-transferases. In contrast, nurse HPGs were enriched in few putative detoxification transcripts and no P450s (Fig. 2). Nurse mandibular glands were enriched in some detoxification transcripts, including P450s, but the subfamilies of enzymes largely differed from those upregulated in foragers, and included members of the *CYP6A*, *CYP49A1* and *CYP6AS* subfamilies. Detoxification transcripts were abundant in the Malpighian tubules and midgut (Table 1, Fig. 1), and forager Malpighian tubules were more highly enriched in transcripts encoding GSTs and P450s than were nurse Malpighian tubules. In the midgut, nurses and foragers differed in the expression of a few detoxification-related transcripts, with transcripts belonging to the *CYP6AS* subfamily upregulated in foragers, while transcripts encoding one *CYP9Q* cytochrome P450 and two variants of a GST were more abundant in nurses (Fig. 2).

**Figure 3:**
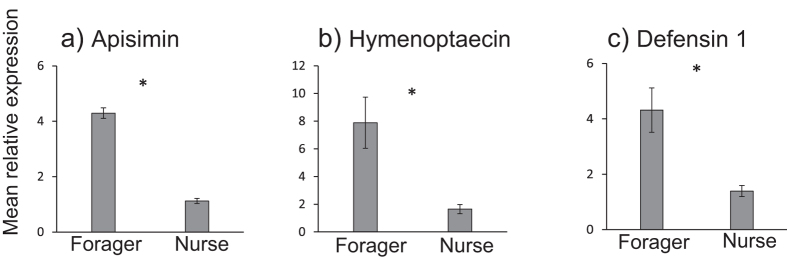
Results of the qPCR analysis of 3 antimicrobial genes that show differential expression in the RNA-Seq analysis between nurses and foragers. Apisimin gene expression (**a**) was measured in the HPG, while defensin-1 (**b**) and hymenoptaecin (**c**) were measured in the Mandibular gland. Mean relative expression (.2^(−∆∆Ct) is shown ± s.e. for the three biological replicates. Asterisks denote that all three genes were differentially expressed between nurses and foragers: Nested ANOVA, *apismin*: *F*_1,12_ = 505.5, *p* < 0.0001, *hymenoptaecin*: *F*_1,12_=682.7, p<0.0001, *defensin-1*: F_1,12_=223.8, p<0.0001.

**Figure 4:**
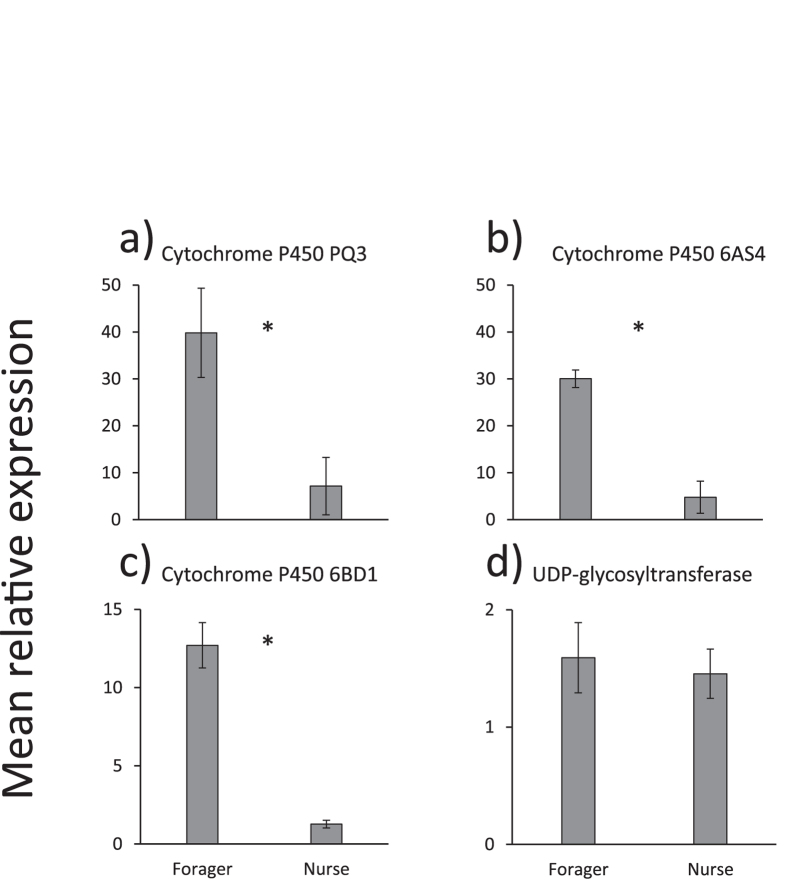
Results of the qPCR analysis of 4 detoxification genes that show differential expression in the RNA-Seq analysis between nurses and foragers. All expression levels were measured in the Mandibular gland. Mean relative expression (.2^(−∆∆Ct) is shown ± s.e. for the three biological replicates. Asterisks denote three P450s that were differentially expressed, while the GST (UDP-glycosyltransferase) was not: Nested-ANOVA, *cytochrome_P450_9Q3*:*F*_1,12_ = 1089.9, *p* < 0.0001, *cytochrome_P450_6BD1*: *F*_1,12_ = 1419.7, p < 0.0001, *cytochrome_P450_6AS4*: *F*_1,12_ = 467.3, *p* < 0.0001, *UDP-glycosyltransferase*: *F*_1,12_ = 0.5, *p* = 0.52.

Additional qPCR was conducted to verify differential expression of a subset of genes identified in the RNA-seq data, listed in Supplementary table S4. Transcripts encoding antimicrobial peptide *apisimin* were more abundant in the forager HPG compared to the nurse HPG (Fig. 3). Similarly, transcripts encoding *defensin-1*, and *hymenoptaecin* were more abundant in the Mandibular gland of foragers compared to nurses, consistent with RNA-seq data (Fig. 3). Three of the four detoxification enzymes examined (*CYP9Q3*, *CYP6BD1*, *CYP6AS4*) were also more abundant in the mandibular glands of foragers compared to the same gland in nurses, while the *UDP-glycosyltransferase* examined was not differentially expressed between worker types in the Mandibular gland (Fig. 4).

## Discussion

The results presented here demonstrate that honey bee foragers exhibit greater expression of genes associated with immune response and detoxification activity than do nurse bees. This difference was particularly pronounced in tissues that mediate nectar processing and social interactions, suggesting a suite of mechanisms by which honey bees may effectively cope with environmental threats acquired while foraging^25^. These differences in expression were supported using two complementary techniques.

Our results highlight the key role of the HPG and mandibular glands in immunity and AMP expression in particular. Previous work has demonstrated expression of AMP genes in these glands^33^, and noted that the function of the HPG changes with worker development^32^,^34^,^35^. We add to this understanding by demonstrating ontogenetic changes in the expression of AMP genes, particularly in the HPG and mandibular glands. In foragers, the HPG plays a key role in the transformation of nectar to honey by producing enzymes that convert sucrose to the monosaccharides characteristic of honey and contribute to the antimicrobial properties of honey. Like previous work, our results document high expression of *α-amylase* and *glucose oxidase* genes, but we also found extremely high expression levels of antimicrobial peptide genes in the HPG. Gene expression levels this high (10^3^–10^6^ RPKM) are rare and restricted to specialized tissues^31^. Extremely high expression and patterns of AMP coexpression with nectar conversion enzymes indicate that the specialized function of the HPG may also include the production of antimicrobial peptides to preserve foraged resources, but further experimental work is necessary to test this hypothesis.

Antimicrobial peptides (AMPs) are effective against brood pathogens frequently encountered within hives^36^, but our data demonstrate that some AMPs are more highly expressed in foragers compared to in-hive nurses. This suggests that both the expression and role of AMPs may change with development^33^. One possibility is that AMPs produced by forager bees may protect nectar against microbial degradation, and this is consistent with the presence of AMPs in honey^37^. We hypothesize that AMPs may be particularly important in reducing microbial activity during early stages of nectar conversion, before low water activity precludes microbial growth^37^. In addition, AMPs were initially detected through their induction by microbial challenge^38^,^39^,^40^. We did not experimentally manipulate bee exposure to microbial effectors in this study, but the differential expression of immune signaling genes in the HPG and mandibular glands (Table 1, Supplementary table S4) suggests that AMP expression in foragers may be induced by exposure to environmental microorganisms^41^,^42^, which are often abundant in floral nectar^7^ or in other foraged resources^43^. Although the activity of specific AMPs against particular microorganisms vary^38^,^40^, complementary activity among peptides may provide an effective defense against a range of microorganisms^39^. However, AMP activity against nectar specialists remains to be tested, as these species often overcome harsh osmotic conditions and damaging peroxides^44^ to ferment sugars in nectar and honey^15^. On the other hand, differential resistance to these peptides may contribute to the formation of ‘core’ and potentially beneficial microbiota^45^. In *Apis*, only a limited number of bacterial taxa can survive in the honey crop^42^ and resistance to AMPs may play a role in structuring this community.

Our study also documented differential expression of putative detoxification enzymes among tissues, as has been previously documented in *Drosophila melanogaster*^46^ and between life stages in *Bombus huntii*^6^. Our results document these patterns in honey bee tissues, but adds that tissues vary in their plasticity in gene expression between life history stages. In our study, many detoxification genes were differentially expressed between nurses and foragers in the HPGs and mandibular glands, compared to few in the Malpighian tubules and midgut. Notably, transcripts encoding P450s from the subfamilies *CYP6AR*, *CYP6AS*, *CYP6BD* and *CYP9Q*–those with demonstrated detoxification activity against phytochemicals^47^,^48^–were among those genes enriched in the forager HPGs and mandibular glands. However, the function of only few P450s have been assayed, so the activity of most of the differentially expressed genes identified here against xenobiotic compounds remain unknown. Nonetheless, we suggest that the activity of those differentially expressed genes should be examined against phytochemicals or pesticides found in nectar and other foraged resources.

The results described here suggest that differential expression of immune signaling genes, antimicrobial effectors and detoxification genes expressed in the HPG and mandibular gland of forager bees may provide a first line of defense against a diverse set of environmentally acquired threats. This study emphasizes the importance of tissue^30^ and role-specific gene expression^6^,^30^, and suggests that multiple tissues, including those involved in nectar processing, should also be examined when assessing honey bee response to pathogens or xenobiotics. Finally, the expression of antimicrobial peptides and effectors of immune response in glands involved in social interactions among bees suggest that social insects, including many pollinators, may employ a wider range of mechanisms against environmentally acquired microorganisms and xenobiotics than previously appreciated^25^.

## Methods

The study makes use of a large RNA-Seq dataset previously analyzed exclusively to explore the role of taxonomically restricted genes in the evolution of novel honey bee traits^31^. Jasper *et al.* (2014) did not explore any of the biological discoveries on which we focus here. We summarize the major methodological information, but complete methods are in^31^. In addition, we used qPCR to validate differential expression of a subset of the genes identified in the RNA-Seq study using an independent dataset.

### Collection of bees and library preparation

Bees were kept at the UC Davis main apiary according to standard beekeeping practices. Three full size colonies were used in the study. Nurse bees were observed with their heads in larval cells for at least 3 seconds, and foragers were identified returning to the hive with pollen. All bees were processed according to previously published methods^30^,^31^. Bees were collected on dry ice, and then stored at −80 °C until use. Tissues (midgut, HPG, mandibular glands, and Malpighian tubules) were dissected within 5 minutes of thawing and total RNA was extracted with the Trizol plus system (Invitrogen). Each bee contributed one tissue type. Tissue from 5–20 individuals was pooled for each biological replicate in accordance to the size of the structure and its RNA yield, and the number of individuals pooled was consistent within a tissue type. On-column digest of DNA using DNAse was performed. Three biological replicates for each tissue for each role (nurses and foragers) were included.

A Nanodrop 1000 was used to check for RNA purity, while a Bioanalyzer 2100 was used to test for both degradation of total RNA and nextgen sequencing library quality. Libraries were made with the NEBNext Illumina RNA-Seq library kit according to the manufacturer’s instructions. Sequencing was performed using the HiSeq 2000 (100 bp paired-end) at the UC Berkeley Vincent Coates Sequencing Center. Reads are available at the NCBI SRA archive (SRP027395, SRP020361, SRP041189). Over 900 million reads total were produced for the four tissues. The number of reads per biological replicate in each tissue is given in Table S6 in Jasper *et al.* (2014).

### Bioinformatics and statistical analyses

Poor quality reads were removed with the FASTX toolkit. Reads with average quality scores less than 25 were removed and the ends of reads clipped to remove low quality base calls. Adaptor contamination was removed with the Cutadapt software package. Tophat (v2.04) with default parameters was used to align reads to the honey bee genome, version 4.5^49^,^50^,^51^. HTSeq^52^ was used to generate counts of reads per gene using the intersection union setting.

To examine the subset of transcribed genes involved in immune response and detoxification, we examined genes with known or suspected antimicrobial activity^39^, immune signaling and response^26^, and detoxification activity^5^,^53^,^54^, see full list in Supplementary table S1 online. Detoxification enzymes included cytochrome P450 monooxygenases (P450s), carboxyl/cholinesterases (CCEs), glutathione-S-transferases (GSTs)^53^. Serine proteases were classified as involved in immune signaling^55^. We also distinguish between P450 monooxygenases with demonstrated detoxification ability in *Apis*, including *CYP6AS* and *CYP9Q* subfamilies^48^, from other P450s with unknown detoxification ability or proposed contribution to sociality in honey bees^53^ through the synthesis or degradation of hormones and pheromones^56^.

Differential expression of transcripts was assessed among tissues (eg. HPG vs all). Within each tissue, transcript abundance was compared between nurses and foragers. Analyses were performed using edgeR^57^ and DE was assessed using the Benjamini-Hochberg (BH) false discovery rate at FDR < 0.05 (Supplementary Figure S2). Here we report results from edgeR, although models implemented in DEseq2^58^ also recovered a similar number and largely overlapping set of DE genes (Supplementary Figure S3).

Although the edgeR analysis was conducted using the full dataset, we focus on the results of genes with known or suspected antimicrobial activity^37^, immune signaling and response^26^, and detoxification activity^5^,^54^,^55^, see full list in Supplementary table S1 online. Detoxification enzymes included cytochrome P450 monooxygenases (P450s), carboxyl/cholinesterases (CCEs), glutathione-S-transferases (GSTs)^54^. Serine proteases were classified as involved in immune signaling^56^. We also distinguish between P450 monooxygenases with demonstrated detoxification ability in *Apis*, including *CYP6AS* and *CYP9Q* subfamilies^49^, from other P450s with unknown detoxification ability or proposed contribution to sociality in honey bees^54^ through the synthesis or degradation of hormones and pheromones^57^.

To examine if honey bee tissues varied in the composition of antimicrobial peptides, signaling molecules or detoxification enzymes transcribed, a permutational MANOVA was conducted. To further assess if particular genes were coexpressed among tissues or between social roles within a tissue, we used principle components analysis (PCA). Nectar conversion enzymes (α-amylase, glucose oxidase, and α-glucosidase) were also included in the PCA to examine their coexpression with immune or detoxification genes. The PCA was performed using the rda function in the vegan package^59^ using log+1 raw transcript abundance of DE genes in each tissue type.

### Quantitative PCR analysis

Real-time PCR validation was carried for 7 genes found to differ between nurses and foragers in the RNA-seq analysis. We chose the three most highly expressed AMPs and four detoxification genes (3 different classes of P450s and one GST), and focused on tissues were differential expression was the greatest. Gene-specific primers spanning exon junctions were designed with NCBI’s primer blast tool (Primer 3 plus the most recent build of the honey bee genome, 4.5). Melt curve and BLAST analysis were used as criteria to determine primer specificity. Primers are listed in Supplementary table S4. Total RNA extracted from three biological replicates (each from a separate colony) of nurse and forager Hypopharyngeal and Mandibular glands were used. The dissections followed the same protocol utilized for the RNA-seq samples (described above), but the bees were from different colonies maintained at the UC Davis apiary. Glands from 10 bees were pooled for each biological replicate^5^. 500 ng of total RNA was used for first-strand cDNA synthesis using the iScript™ Reverse Transcription System. This system uses an optimized blend of oligo(dT) and random primers to target an unbiased representation of target genes. The qPCR assays were performed using SsoAdvanced™ Universal SYBR® Green Supermix (Bio-rad, Hercules, CA) in a CFX96 Touch Real-Time PCR Detection thermal cycler (Bio-Rad, Hercules, CA). Cycling conditions were 95 °C for 30 seconds, 40 cycles of 95 °C for 5 seconds, followed by an annealing/extension phase at 60 °C for 30 seconds. The reaction was concluded with a melt curve analysis going from 65 °C to 95 °C in 0.5 °C increments at 5 seconds per step. Three technical replicates were performed for each biological replicate. Data were analyzed using the standard ΔΔCt method and target gene mRNA expression levels were normalized to an established reference gene’s mRNA levels (*eukaryotic translation initiation factor 3 subunit C (eIF3-S8)*), which was previously validated for qPCR normalization in honey bees^5^. Nested ANOVA was used to examine expression differences between nurses and foragers for each gene separately (replicate nested within worker type), and analyses were performed using Minitab.

Data accessibility: The raw RNA-seq data are available at the NCBI SRA archive (SRP027395,SRP020361, SRP041189).

## Additional Information

**How to cite this article**: Vannette, R. L. *et al.* Forager bees (*Apis mellifera*) highly express immune and detoxification genes in tissues associated with nectar processing. *Sci. Rep.* **5**, 16224; doi: 10.1038/srep16224 (2015).

## Supplementary Material

Supplementary Information

## Figures and Tables

**Table 1:** Genes differentially expressed among *Apis mellifera* tissues, where 1 indicates greater abundance in the focal tissue compared to the average of all tissues.

Class	Gene name or subfamily	HPG	Tissue type		
			Mandibular gland	Malpighian tubules	Midgut
AMPs					
	*apisimin*	1	0	0	0
	*defensin-1*	1	0	0	0
	*hymenoptaecin*	1	0	0	0
	Total	3	0	0	0
CCEs		0	0	3	1
GSTs		1	0	4	5
Laccases		0	0	1	0
Lysozymes		0	0	0	1
P450s					
	*CYP6AS*	0	0	4	1
	*CYP9Q*	0	0	2	0
	other CYP450s	1	0	4	3
	Total	1	0	10	4
Phagocytosis		0	1	0	0
Serine proteases		3	0	0	3
Signaling		3	1	3	5

Differential expression was analyzed using edgeR, with FDR < 0.05. Numbers in bold indicate gene class totals. Abbreviations for gene classes include antimicrobial peptides (AMPs), carboxyl/cholinesterases (CCEs), glutathione-S-transferases (GSTs), and cytochrome P450 monooxygenases (P450s). See methods for complete details. A list of all genes examined and the full names of differentially expressed genes are included in Supplementary table S1.
