# Supplementary material for: Forager bees (Apis mellifera) highly express immune and detoxification genes in tissues associated with nectar processing
Source: Sci Rep. 2015 Nov 9;5:16224. doi: 10.1038/srep16224 (PMC4637902; doi:10.1038/srep16224)
Supplement: Supplementary Information [file srep16224-s1.doc]

Supplementary information for “Forager bees (*Apis mellifera*) highly express immune and detoxification genes in tissues associated with nectar processing”

Authors: RL Vannette, A Mohamed & BR Johnson

**Supplementary table S1** List of genes with putative antimicrobial or detoxification functions, taken from supplementary materials provided in Claudianos et al (2006), Evans et al (2006), and Mao et al (2011). Beebase IDs (if available) were mapped to Genbank IDs using DAVID online conversion tool. Class abbreviations include antimicrobial peptide or peptides with similar structure (AMP), carboxyl/cholinesterases (CCE), glutathione-S-transferases (GSTs) and cytochrome P450 monooxygenases (P450s).

| **Genbank.ID** | **BeeBase** | **Gene.Name** | **Gene.Family.Pathway** | **Class** |
| --- | --- | --- | --- | --- |
| AF442147 | GB18323 | abaecin | AMP | AMP |
| AY588474 | GB10036 | Defensin-2 | AMP | AMP |
| DB729862 | GB19392 | Defensin-1 | AMP | AMP |
| DB758435 | GB19392 | Defensin-1 | AMP | AMP |
| HP463277 | GB17538 | Hymenoptaecin | AMP | AMP |
| NM_001011582 | GB19468 | apisimin | AMP | AMP |
| NM_001011615 | GB17538 | Hymenoptaecin | AMP | AMP |
| NM_001011616 | GB19392 | Defensin-1 | AMP | AMP |
| U15954 | GB18323 | abaecin | AMP | AMP |
| AY055108 | GB19468 | apisimin | AMP | AMP |
| FJ546166 | GB17538 | Hymenoptaecin | AMP | AMP |
| NM_001011582 | NA | apisimin | AMP | AMP |
| NM_001011613 | NA | apidaecin_1 | AMP | AMP |
| NM_001011615 | NA | hymenoptaecin | AMP | AMP |
| NM_001011616 | NA | Defensin-1 | AMP | AMP |
| NM_001011638 | GB10036 | Defensin-2 | AMP | AMP |
| NM_001085344 | NA | apidermin_3 | AMP | AMP |
| NM_001085345 | NA | apidermin_1 | AMP | AMP |
| NM_001174144 | NA | apidermin-like | AMP | AMP |
| U15955 | GB19392 | Defensin-1 | AMP | AMP |
| XM_006569641 | GB18323 | abaecin | AMP | AMP |
| XM_006572699 | NA | apidaecin | AMP | AMP |
| AF213011 | GB19866 | esterase | CCE | CCE |
| AF213012 | GB14873 | AChE-2 | CCE | CCE |
| AY526235 | GB15327 | JH esterase | CCE | CCE |
| EU564833 | GB10820 | esterase | CCE | CCE |
| FJ580048 | GB18720 | NLG-1 | CCE | CCE |
| FJ580049 | GB18290 | NLG-3 | CCE | CCE |
| FJ580050 | GB18836 | NLG-4 | CCE | CCE |
| FJ580052 | GB10066 | NLG-2 | CCE | CCE |
| NM_001040230 | GB14873 | AChE-2 | CCE | CCE |
| NM_001134947 | GB19866 | esterase | CCE | CCE |
| NM_001142609 | GB15030 | esterase | CCE | CCE |
| NM_001145736 | GB18290 | NLG-3 | CCE | CCE |
| NM_001145737 | GB18836 | NLG-4 | CCE | CCE |
| XM_001121481 | GB14476 | esterase | CCE | CCE |
| XM_006560167 | GB19866 | esterase | CCE | CCE |
| XM_006561836 | GB18720 | NLG-1 | CCE | CCE |
| XM_006561837 | GB18720 | NLG-1 | CCE | CCE |
| XM_006563874 | GB15327 | esterase | CCE | CCE |
| XM_006564243 | GB15030 | esterase | CCE | CCE |
| XM_006564295 | GB13591 | esterase | CCE | CCE |
| XM_006564296 | GB13591 | esterase | CCE | CCE |
| XM_006564399 | GB13602 | esterase | CCE | CCE |
| XM_006566684 | GB12309 | gli | CCE | CCE |
| XM_006566867 | GB11403 | esterase | CCE | CCE |
| XM_006571491 | GB10066 | NLG-2 | CCE | CCE |
| XM_006571493 | GB10066 | NLG-2 | CCE | CCE |
| XM_391943 | GB11403 | esterase | CCE | CCE |
| XM_393293 | GB16889 | esterase | CCE | CCE |
| XM_393670 | GB15536 | esterase | CCE | CCE |
| XM_393751 | GB18414 | AChE-1 | CCE | CCE |
| XM_394697 | GB13591 | esterase | CCE | CCE |
| XM_396693 | GB18901 | esterase | CCE | CCE |
| XM_396706 | GB12309 | gli | CCE | CCE |
| XR_119930 | GB10854 | esterase | CCE | CCE |
| XR_409958 | GB13602 | esterase | CCE | CCE |
| AB083009 | GB15327 | esterase | CCE | CCE |
| AB181702 | GB14873 | AChE-2 | CCE | CCE |
| HP574572 | GB15030 | esterase | CCE | CCE |
| NM_001126244 | GB10820 | esterase | CCE | CCE |
| XM_006561555 | GB18836 | NLG-4 | CCE | CCE |
| XM_006564244 | NA | esterase | CCE | CCE |
| XM_006564297 | GB13591 | esterase | CCE | CCE |
| XM_006564398 | GB13602 | esterase | CCE | CCE |
| XM_006568066 | GB18660 | esterase | CCE | CCE |
| XM_006570334 | GB18414 | AChE-1 | CCE | CCE |
| XM_391995 | GB19830 | nrt | CCE | CCE |
| XM_392698 | GB11064 | esterase | CCE | CCE |
| FJ374871 | GB14372 | GSTS4 | GSTs | GST |
| NM_001160270 | GB16959 | GSTS1 | GSTs | GST |
| XM_001121652 | GB10566 | GST | GSTs | GST |
| XM_006560503 | GB14372 | GSTS4 | GSTs | GST |
| XM_006560504 | GB14372 | GSTS4 | GSTs | GST |
| XM_006563329 | GB18045 | GSTD1 | GSTs | GST |
| XM_006563331 | GB18045 | GSTD1 | GSTs | GST |
| XM_006563333 | GB18045 | GSTD1 | GSTs | GST |
| XM_006572043 | GB16959 | GSTS1 | GSTs | GST |
| XM_394313 | GB12371 | GST | GSTs | GST |
| XM_624498 | GB11466 | GSTO1 | GSTs | GST |
| XM_624689 | GB12047 | GSTT1 | GSTs | GST |
| AY620822 | GB18045 | GSTD1 | GSTs | GST |
| FJ873798 | GB16959 | GSTS1 | GSTs | GST |
| XM_001121779 | NA | GST | GSTs | GST |
| XM_001121779 | NA | GST | GSTs | GST |
| XM_001122087 | GB19254 | GSTS3 | GSTs | GST |
| XM_001123301 | NA | GST | GSTs | GST |
| XM_001123301 | NA | GST | GSTs | GST |
| XM_006560047 | NA | GST | GSTs | GST |
| XM_006563332 | NA | GST | GSTs | GST |
| XM_006563668 | NA | GST | GSTs | GST |
| XM_006564397 | NA | GST | GSTs | GST |
| XM_006566233 | NA | GST | GSTs | GST |
| XM_006569632 | GB11466 | GSTO1 | GSTs | GST |
| XM_006572045 | NA | GST | GST | GST |
| XM_392319 | NA | glucuronosyltransferase | GST | GST |
| XM_392727 | NA | glucuronosyltransferase | GST | GST |
| XM_394494 | NA | UDP-glycosyltransferase | GST | GST |
| XM_394562 | GB17672 | GSTZ1 | GSTs | GST |
| XM_394772 | NA | UDP-glycosyltransferase | GST | GST |
| XM_396494 | NA | UDP-glycosyltransferase | GST | GST |
| XM_001120790 | NA | laccase-1 | laccase | laccase |
| XM_006562254 | NA | laccase-2 | laccase | laccase |
| XM_625186 | NA | laccase-5-like | laccase | laccase |
| XM_393161 | GB19988 | Lys-3 | Lysozyme | Lysozyme |
| XM_001120136 | GB15106 | Lys-2 | Lysozyme | Lysozyme |
| XM_003249626 | GB10231 | Lys-1 | Lysozyme | Lysozyme |
| XM_006571783 | GB19988 | Lys-3 | Lysozyme | Lysozyme |
| AB558125 | GB13998 | 314A1 | P450s | P450s |
| AB558127 | GB16447 | 315A1 | P450s | P450s |
| AB558128 | GB15545 | 302A1 | P450s | P450s |
| AB558129 | GB12311 | 306A1 | P450s | P450s |
| AB604029 | GB18019 | 307B1 | P450s | P450s |
| DQ232888 | GB17434 | 6AS5 | P450s | P450s |
| DQ244074 | GB13998 | 314A1 | P450s | P450s |
| HP460695 | GB14913 | 6AS15 | P450s | P450s |
| HP469969 | GB15409 | 6AQ1 | P450s | P450s |
| HP477347 | GB15681 | 6AS3 | P450s | P450s |
| HP477348 | GB15681 | 6AS3 | P450s | P450s |
| HP502655 | GB14913 | 6AS15 | P450s | P450s |
| HP573236 | GB14913 | 6AS15 | P450s | P450s |
| HP576826 | GB18019 | 307B1 | P450s | P450s |
| NM_001040233 | GB11973 | 4G11 | P450s | P450s |
| XM_001119928 | GB10856 | 342A1 | P450s | P450s |
| XM_001121037 | GB14594 | 6AS10 | P450s | P450s |
| XM_001121195 | GB12608 | 334A1 | P450s | P450s |
| XM_001121960 | GB19113 | 6AS14 | P450s | P450s |
| XM_006557825 | GB12311 | 306A1 | P450s | P450s |
| XM_006558617 | GB18872 | 303A1 | P450s | P450s |
| XM_006559341 | GB11973 | 4G11 | P450s | P450s |
| XM_006561859 | GB12608 | 334A1 | P450s | P450s |
| XM_006562301 | GB19820 | 9Q1 | P450s | P450s |
| XM_006563932 | GB16447 | 315A1 | P450s | P450s |
| XM_006564436 | GB19306 | 6BD1 | P450s | P450s |
| XM_006564999 | GB12136 | 6AS12 | P450s | P450s |
| XM_006565001 | GB18052 | 6AS7 | P450s | P450s |
| XM_006565014 | GB11754 | 6AS8 | P450s | P450s |
| XM_006565072 | GB15681 | 6AS3 | P450s | P450s |
| XM_006565073 | GB11027 | 6AS11 | P450s | P450s |
| XM_006565328 | GB10466 | 6BC1 | P450s | P450s |
| XM_006566840 | GB15409 | 6AQ1 | P450s | P450s |
| XM_006566842 | GB15409 | 6AQ1 | P450s | P450s |
| XM_006570168 | GB13998 | 314A1 | P450s | P450s |
| XM_006571856 | GB14915 | 343A1 | P450s | P450s |
| XM_006571857 | GB14915 | 343A1 | P450s | P450s |
| XM_392000 | GB17793 | 9Q2 | P450s | P450s |
| XM_393974 | GB18019 | 307B1 | P450s | P450s |
| XM_395671 | GB15793 | 6AS4 | P450s | P450s |
| XM_396534 | GB11754 | 6AS8 | P450s | P450s |
| XM_397170 | GB11406 | 301A1 | P450s | P450s |
| XM_623359 | GB17588 | 6AR1 | P450s | P450s |
| XM_623572 | GB15634 | 15A1 | P450s | P450s |
| XM_623618 | GB11943 | 305D1 | P450s | P450s |
| XM_624017 | GB10668 | 6AS17 | P450s | P450s |
| AB558130 | GB18019 | 307B1 | P450s | P450s |
| DQ244075 | GB11973 | 4G11 | P450s | P450s |
| HP460696 | GB14913 | 6AS15 | P450s | P450s |
| HP502489 | GB16447 | 315A1 | P450s | P450s |
| HP509951 | GB13998 | 314A1 | P450s | P450s |
| NM_001040234 | GB17434 | 6AS5 | P450s | P450s |
| NM_001278333 | NA | 4aa1-like | P450s | P450s |
| XM_001119981 | GB19797 | 336A1 | P450s | P450s |
| XM_001120976 | GB12885 | 6AS16P | P450s | P450s |
| XM_001121037 | NA | AS10 | P450s | P450s |
| XM_001123306 | NA | 6A13-like | P450s | P450s |
| XM_006557824 | GB12311 | 306A1 | P450s | P450s |
| XM_006562300 | GB19967 | 9Q3 | P450s | P450s |
| XM_006562301 | NA | 9Q1 | P450s | P450s |
| XM_006562324 | GB13748 | 9S1 | P450s | P450s |
| XM_006562483 | NA | 49A1 | P450s | P450s |
| XM_006564367 | GB10905 | 4AZ1 | P450s | P450s |
| XM_006564436 | NA | 6BD1 | P450s | P450s |
| XM_006565000 | GB10668 | 6AS17 | P450s | P450s |
| XM_006565002 | GB18052 | 6AS7 | P450s | P450s |
| XM_006565013 | GB11754 | 6AS8 | P450s | P450s |
| XM_006565073 | NA | 6AS11 | P450s | P450s |
| XM_006567923 | GB18743 |  | P450s | P450s |
| XM_006570169 | NA | 314A1 | P450s | P450s |
| XM_006572405 | NA | 6A13-like | P450s | P450s |
| XM_006572724 | NA | 6a21-like | P450s | P450s |
| XM_392000 | NA | 9Q2 | P450s | P450s |
| XM_393885 | GB14343 | 18A1 | P450s | P450s |
| XM_393969 | GB16803 | 9R1 | P450s | P450s |
| XM_395085 | GB19197 | 6AS2 | P450s | P450s |
| XM_396751 | GB16899 | 6AS1 | P450s | P450s |
| XM_396849 | GB19113 | 6AS14 | P450s | P450s |
| XM_397347 | GB12136 | 6AS12 | P450s | P450s |
| XM_623359 | NA | 6AR1 | P450s | P450s |
| XM_623424 | NA | 6AS13 | P450s | P450s |
| XM_623592 | NA | 6AS15 | P450s | P450s |
| XM_624023 | GB14915 | 343A1 | P450s | P450s |
| XM_624792 | GB14612 | 6BE1 | P450s | P450s |
| XM_006561042 | GB13979 | NimC2 | Phagocytosis | Phagocytosis |
| XM_394098 | GB13979 | NimC2 | Phagocytosis | Phagocytosis |
| XM_001120328 | GB12883 | NimA | Phagocytosis | Phagocytosis |
| XM_006561041 | GB13979 | NimC2 | Phagocytosis | Phagocytosis |
| XM_006561053 | GB14645 | NimC1 | Phagocytosis | Phagocytosis |
| AY127579 | GB11552 | SP34 | serine proteases | Serine protease |
| DQ288392 | GB14366 | cSPH39 | serine proteases | Serine protease |
| HP523017 | GB14774 | SP47 | serine proteases | Serine protease |
| NM_001040231 | GB14366 | cSPH39 | serine proteases | Serine protease |
| XM_001120029 | GB11297 | SP31 | serine proteases | Serine protease |
| XM_001120043 | GB17927 | cSP10 | serine proteases | Serine protease |
| XM_001120112 | GB16038 | SPH57 | serine proteases | Serine protease |
| XM_001120594 | GB15317 | SP49 | serine proteases | Serine protease |
| XM_001120817 | GB15254 | cSPH55 | serine proteases | Serine protease |
| XM_001121032 | GB14044 | cSP14 | serine proteases | Serine protease |
| XM_001122073 | GB11511 | SP32 | serine proteases | Serine protease |
| XM_001122095 | GB14603 | SP17 | serine proteases | Serine protease |
| XM_001122651 | GB14001 | cSPH50 | serine proteases | Serine protease |
| XM_006557812 | GB14077 | cSP6 | serine proteases | Serine protease |
| XM_006557813 | GB14077 | cSP6 | serine proteases | Serine protease |
| XM_006557814 | GB14077 | cSP6 | serine proteases | Serine protease |
| XM_006557831 | GB13019 | SPH56 | serine proteases | Serine protease |
| XM_006558187 | GB14366 | cSPH39 | serine proteases | Serine protease |
| XM_006558239 | GB18944 | SPH37 | serine proteases | Serine protease |
| XM_006558885 | GB18178 | SP15 | serine proteases | Serine protease |
| XM_006558886 | GB18178 | SP15 | serine proteases | Serine protease |
| XM_006558887 | GB18178 | SP15 | serine proteases | Serine protease |
| XM_006559049 | GB14774 | SP47 | serine proteases | Serine protease |
| XM_006559330 | GB14309 | cSP33 | serine proteases | Serine protease |
| XM_006559331 | GB14309 | cSP33 | serine proteases | Serine protease |
| XM_006559333 | GB19719 | cSP25 | serine proteases | Serine protease |
| XM_006559676 | GB19590 | SP20 | serine proteases | Serine protease |
| XM_006560557 | GB11698 | cSP3 | serine proteases | Serine protease |
| XM_006561010 | GB12253 | SP16 | serine proteases | Serine protease |
| XM_006561435 | GB12379 | SP48 | serine proteases | Serine protease |
| XM_006561660 | GB15980 | SPH54 | serine proteases | Serine protease |
| XM_006561661 | GB15980 | SPH54 | serine proteases | Serine protease |
| XM_006561662 | GB15980 | SPH54 | serine proteases | Serine protease |
| XM_006562026 | GB17654 | SP45 | serine proteases | Serine protease |
| XM_006562027 | GB17654 | SP45 | serine proteases | Serine protease |
| XM_006562691 | GB12538 | SP23 | serine proteases | Serine protease |
| XM_006563255 | GB16367 | SP46 | serine proteases | Serine protease |
| XM_006563256 | GB16367 | SP46 | serine proteases | Serine protease |
| XM_006563693 | GB10943 | cSPH41 | serine proteases | Serine protease |
| XM_006563716 | GB15254 | cSPH55 | serine proteases | Serine protease |
| XM_006564467 | GB19649 | SP30 | serine proteases | Serine protease |
| XM_006564468 | GB19649 | SP30 | serine proteases | Serine protease |
| XM_006564910 | GB18450 | cSP26 | serine proteases | Serine protease |
| XM_006565724 | GB17345 | SPH19 | serine proteases | Serine protease |
| XM_006565725 | GB17345 | SPH19 | serine proteases | Serine protease |
| XM_006567140 | GB13489 | SP28 | serine proteases | Serine protease |
| XM_006567184 | GB16220 | cSP21 | serine proteases | Serine protease |
| XM_006567383 | GB14001 | cSPH50 | serine proteases | Serine protease |
| XM_006570203 | GB14247 | cSP2 | serine proteases | Serine protease |
| XM_006570929 | GB19292 | SPH52 | serine proteases | Serine protease |
| XM_006571576 | GB14044 | cSP14 | serine proteases | Serine protease |
| XM_006572557 | GB11588 | SP27 | serine proteases | Serine protease |
| XM_392080 | GB15453 | SP44 | serine proteases | Serine protease |
| XM_392728 | GB18944 | SPH37 | serine proteases | Serine protease |
| XM_393127 | GB13263 | SP40 | serine proteases | Serine protease |
| XM_393727 | GB16214 | SP38 | serine proteases | Serine protease |
| XM_394005 | GB18450 | cSP26 | serine proteases | Serine protease |
| XM_394101 | GB12253 | SP16 | serine proteases | Serine protease |
| XM_394370 | GB19846 | SP36 | serine proteases | Serine protease |
| XM_394832 | GB12300 | SP5 | serine proteases | Serine protease |
| XM_397087 | GB13791 | SP22 | serine proteases | Serine protease |
| XM_623147 | GB11298 | cSPH42 | serine proteases | Serine protease |
| XM_623896 | GB13019 | SPH56 | serine proteases | Serine protease |
| XR_410879 | GB16220 | cSP21 | serine proteases | Serine protease |
| HP564584 | GB14774 | SP47 | serine proteases | Serine protease |
| NM_001011584 | GB11552 | SP34 | serine proteases | Serine protease |
| XM_001120508 | GB14644 | SP29 | serine proteases | Serine protease |
| XM_001120871 | GB12538 | SP23 | serine proteases | Serine protease |
| XM_001121888 | GB18767 | SP8 | serine proteases | Serine protease |
| XM_001122420 | GB15640 | SP13 | serine proteases | Serine protease |
| XM_001123170 | GB18732 | SP9 | serine proteases | Serine protease |
| XM_003249197 | GB11298 | cSPH42 | serine proteases | Serine protease |
| XM_006557830 | GB13019 | SPH56 | serine proteases | Serine protease |
| XM_006558188 | GB14366 | cSPH39 | serine proteases | Serine protease |
| XM_006558238 | GB18944 | SPH37 | serine proteases | Serine protease |
| XM_006558368 | GB13397 | SPH51 | serine proteases | Serine protease |
| XM_006559332 | GB14309 | cSP33 | serine proteases | Serine protease |
| XM_006559334 | GB19719 | cSP25 | serine proteases | Serine protease |
| XM_006561011 | GB12253 | SP16 | serine proteases | Serine protease |
| XM_006561436 | GB12379 | SP48 | serine proteases | Serine protease |
| XM_006563257 | GB16367 | SP46 | serine proteases | Serine protease |
| XM_006564466 | GB19649 | SP30 | serine proteases | Serine protease |
| XM_006565726 | GB17345 | SPH19 | serine proteases | Serine protease |
| XM_006567183 | GB16220 | cSP21 | serine proteases | Serine protease |
| XM_006570204 | GB16147 | cSP1 | serine proteases | Serine protease |
| XM_006570807 | GB11297 | SP31 | serine proteases | Serine protease |
| XM_006570928 | GB19292 | SPH52 | serine proteases | Serine protease |
| XM_006571406 | GB14233 | SP24 | serine proteases | Serine protease |
| XM_006571575 | GB14044 | cSP14 | serine proteases | Serine protease |
| XM_392669 | GB13489 | SP28 | serine proteases | Serine protease |
| XM_624677 | GB16021 | SP35 | serine proteases | Serine protease |
| XM_624881 | GB15702 | SPH53 | serine proteases | Serine protease |
| XM_625048 | GB17145 | cSP7 | serine proteases | Serine protease |
| AY242387 | GB18313 | PPO | PPO | Signaling |
| AY937243 | GB15177 | 18-w | Toll/TLR | Signaling |
| BI516893 | GB19301 | PGRP-S2 | PGRP | Signaling |
| DB756241 | GB19452 | B-gluc1 | GNBPs | Signaling |
| DB770993 | GB19452 | B-gluc1 | GNBPs | Signaling |
| FJ546112 | GB17879 | PGRP-S3 | PGRP | Signaling |
| FJ546117 | GB19301 | PGRP-S2 | PGRP | Signaling |
| GU358193 | GB17330 | CTL5 | C-lectin domain | Signaling |
| HP501815 | GB12933 | IGFn3-14 | IG Superfamily Genes | Signaling |
| HP516643 | GB12933 | IGFn3-14 | IG Superfamily Genes | Signaling |
| HP517768 | GB19498 | dUbc13 | IMD | Signaling |
| HP525159 | GB12933 | IGFn3-14 | IG Superfamily Genes | Signaling |
| HP572815 | GB19498 | dUbc13 | IMD | Signaling |
| HP574897 | GB19683 | AmSCR-B10 | Scav. Receptor B | Signaling |
| HQ824710 | GB11358 | IGFn3-2 | IG Superfamily Genes | Signaling |
| NM_001011627 | GB18313 | PPO | PPO | Signaling |
| NM_001163712 | GB10655 | cact-1 | Toll/TLR | Signaling |
| NM_001163714 | GB19452 | B-gluc1 | GNBPs | Signaling |
| NM_001163715 | GB17879 | PGRP-S3 | PGRP | Signaling |
| NM_001171006 | GB19066 | dorsal-1A | Toll/TLR | Signaling |
| NM_001171006 | GB19537 | Dorsal-1B | Toll/TLR | Signaling |
| XM_001120043 | GB17927 | PSH LIKE | Toll/TLR | Signaling |
| XM_001120347 | GB11717 | CTL1 | C-lectin domain | Signaling |
| XM_001120619 | GB17106 | IKKgamma-kenny | IMD | Signaling |
| XM_001121032 | GB14044 | PSH LIKE | Toll/TLR | Signaling |
| XM_001121110 | GB11902 | Scabrous | Fibrinogen | Signaling |
| XM_001121229 | GB15684 | tube | Toll/TLR | Signaling |
| XM_001121634 | GB19961 | B-gluc2 | GNBPs | Signaling |
| XM_001122664 | GB18650 | Tab | IMD | Signaling |
| XM_006557239 | GB16401 | MAPK(basket) | MAPK | Signaling |
| XM_006557244 | GB16401 | MAPK(basket) | MAPK | Signaling |
| XM_006557245 | GB16401 | MAPK(basket) | MAPK | Signaling |
| XM_006557980 | GB17167 | hem | JNK | Signaling |
| XM_006558045 | GB13808 | CTL12 | C-lectin domain | Signaling |
| XM_006558046 | GB13808 | CTL12 | C-lectin domain | Signaling |
| XM_006558108 | GB15987 | IGFn3-3 | IG Superfamily Genes | Signaling |
| XM_006558115 | GB15987 | IGFn3-3 | IG Superfamily Genes | Signaling |
| XM_006558641 | GB14382 | CTL8 | C-lectin domain | Signaling |
| XM_006558643 | GB14382 | CTL8 | C-lectin domain | Signaling |
| XM_006558985 | GB11792 | CTL6 | C-lectin domain | Signaling |
| XM_006558986 | GB11792 | CTL6 | C-lectin domain | Signaling |
| XM_006560118 | GB14520 | IGFn3-12 | IG Superfamily Genes | Signaling |
| XM_006560120 | GB14520 | IGFn3-12 | IG Superfamily Genes | Signaling |
| XM_006560121 | GB14520 | IGFn3-12 | IG Superfamily Genes | Signaling |
| XM_006560439 | GB12344 | MyD88 | Toll/TLR | Signaling |
| XM_006560440 | GB12344 | MyD88 | Toll/TLR | Signaling |
| XM_006560441 | GB12344 | MyD88 | Toll/TLR | Signaling |
| XM_006560641 | GB13360 | lox2-like | Scav. Receptor A | Signaling |
| XM_006561055 | GB18362 | D-PIAS | JakSTAT | Signaling |
| XM_006561056 | GB18362 | D-PIAS | JakSTAT | Signaling |
| XM_006561451 | GB19925 | AmSCR-C | Scav. Receptor C | Signaling |
| XM_006562123 | GB16060 | IGFn3-9 | IG Superfamily Genes | Signaling |
| XM_006562219 | GB13742 | relish | IMD | Signaling |
| XM_006562221 | GB13742 | relish | IMD | Signaling |
| XM_006562507 | GB10539 | TRAF2 | Toll/TLR | Signaling |
| XM_006562602 | GB20122 | CTL4 | C-lectin domain | Signaling |
| XM_006562633 | GB12830 | AmSCR-B4 | Scav. Receptor B | Signaling |
| XM_006562636 | GB12830 | AmSCR-B4 | Scav. Receptor B | Signaling |
| XM_006562637 | GB12830 | AmSCR-B4 | Scav. Receptor B | Signaling |
| XM_006562691 | GB12538 | GRAAL/Tequila-like | Scav. Receptor A | Signaling |
| XM_006562870 | GB15549 | AmSCR-B7 | Scav. Receptor B | Signaling |
| XM_006562871 | GB15549 | AmSCR-B7 | Scav. Receptor B | Signaling |
| XM_006563920 | GB10912 | IGFn3-6 | IG Superfamily Genes | Signaling |
| XM_006564043 | GB19301 | PGRP-S2 | PGRP | Signaling |
| XM_006564054 | GB15371 | PGRP-S1 | PGRP | Signaling |
| XM_006564153 | GB12212 | Kay | JNK | Signaling |
| XM_006564154 | GB12212 | Kay | JNK | Signaling |
| XM_006564155 | GB12212 | Kay | JNK | Signaling |
| XM_006564415 | GB13522 | MAPKKK9 | MAPK | Signaling |
| XM_006564467 | GB19649 | Corin-like | Scav. Receptor A | Signaling |
| XM_006564468 | GB19649 | Corin-like | Scav. Receptor A | Signaling |
| XM_006564518 | GB16711 | Hemolectin | Cellular response | Signaling |
| XM_006564520 | GB16711 | Hemolectin | Cellular response | Signaling |
| XM_006565161 | GB13813 | AmSCR-B5 | Scav. Receptor B | Signaling |
| XM_006565162 | GB13813 | AmSCR-B5 | Scav. Receptor B | Signaling |
| XM_006565441 | GB12605 | TEP7 | TEP | Signaling |
| XM_006565455 | GB18032 | Dorsal-2 | Toll/TLR | Signaling |
| XM_006565669 | GB15344 | IGFn3-15 | IG Superfamily Genes | Signaling |
| XM_006565672 | GB15344 | IGFn3-15 | IG Superfamily Genes | Signaling |
| XM_006565777 | GB18650 | Tab | IMD | Signaling |
| XM_006566589 | GB16970 | Pellino | Toll/TLR | Signaling |
| XM_006566590 | GB16970 | Pellino | Toll/TLR | Signaling |
| XM_006566633 | GB14265 | CTL2 | C-lectin domain | Signaling |
| XM_006566935 | GB10506 | AmSCR-B1 | Scav. Receptor B | Signaling |
| XM_006566937 | GB10506 | AmSCR-B1 | Scav. Receptor B | Signaling |
| XM_006566997 | GB19066 | dorsal-1A | Toll/TLR | Signaling |
| XM_006566997 | GB19537 | Dorsal-1B | Toll/TLR | Signaling |
| XM_006567001 | GB19537 | Dorsal-1B | Toll/TLR | Signaling |
| XM_006567107 | GB10655 | cact-1 | Toll/TLR | Signaling |
| XM_006567108 | GB10655 | cact-1 | Toll/TLR | Signaling |
| XM_006567136 | GB13261 | IGFn3-5 | IG Superfamily Genes | Signaling |
| XM_006567137 | GB13261 | IGFn3-5 | IG Superfamily Genes | Signaling |
| XM_006567139 | GB13261 | IGFn3-5 | IG Superfamily Genes | Signaling |
| XM_006567220 | GB19916 | AmSCR-B9 | Scav. Receptor B | Signaling |
| XM_006567221 | GB19916 | AmSCR-B9 | Scav. Receptor B | Signaling |
| XM_006567689 | GB16422 | Hopscotch | JakSTAT | Signaling |
| XM_006567690 | GB16422 | Hopscotch | JakSTAT | Signaling |
| XM_006567802 | GB11846 | IGFn3-7 | IG Superfamily Genes | Signaling |
| XM_006567953 | GB14317 | IGFn3-8 | IG Superfamily Genes | Signaling |
| XM_006568755 | GB11358 | IGFn3-2 | IG Superfamily Genes | Signaling |
| XM_006569284 | GB11807 | IGFn3-10 | IG Superfamily Genes | Signaling |
| XM_006569286 | GB11807 | IGFn3-10 | IG Superfamily Genes | Signaling |
| XM_006569289 | GB11807 | IGFn3-10 | IG Superfamily Genes | Signaling |
| XM_006569290 | GB12490 | IGFn3-11 | IG Superfamily Genes | Signaling |
| XM_006569291 | GB12490 | IGFn3-11 | IG Superfamily Genes | Signaling |
| XM_006569294 | GB12490 | IGFn3-11 | IG Superfamily Genes | Signaling |
| XM_006569296 | GB14314 | AmSCR-B6 | Scav. Receptor B | Signaling |
| XM_006570603 | GB18949 | SOCS | JakSTAT | Signaling |
| XM_006570669 | GB17018 | Angiopoietin | Fibrinogen | Signaling |
| XM_006570670 | GB17018 | Angiopoietin | Fibrinogen | Signaling |
| XM_006570965 | GB11563 | TEPB | TEP | Signaling |
| XM_006571576 | GB14044 | PSH LIKE | Toll/TLR | Signaling |
| XM_006571765 | GB18789 | TEPA | TEP | Signaling |
| XM_006571808 | GB15050 | CTL9 | C-lectin domain | Signaling |
| XM_006571810 | GB15050 | CTL9 | C-lectin domain | Signaling |
| XM_006572294 | GB14664 | Tak1 | IMD | Signaling |
| XM_392321 | GB19916 | AmSCR-B9 | Scav. Receptor B | Signaling |
| XM_392752 | GB19683 | AmSCR-B10 | Scav. Receptor B | Signaling |
| XM_393637 | GB14265 | CTL2 | C-lectin domain | Signaling |
| XM_393713 | GB10640 | Toll-8/Trex | Toll/TLR | Signaling |
| XM_393774 | GB18049 | CTL3 | C-lectin domain | Signaling |
| XM_394552 | GB16060 | IGFn3-9 | IG Superfamily Genes | Signaling |
| XM_395180 | GB18032 | Dorsal-2 | Toll/TLR | Signaling |
| XM_395296 | GB11846 | IGFn3-7 | IG Superfamily Genes | Signaling |
| XM_395686 | GB18324 | Galectin-2 | Galectin | Signaling |
| XM_395991 | GB17012 | NEC LIKE | Toll/TLR | Signaling |
| XM_396277 | GB20122 | CTL4 | C-lectin domain | Signaling |
| XM_396819 | GB11057 | lap2 | IMD | Signaling |
| XM_396852 | GB11743 | AmSCR-B2 | Scav. Receptor B | Signaling |
| XM_396937 | GB14720 | Mik2 | Toll/TLR | Signaling |
| XM_397010 | GB15344 | IGFn3-15 | IG Superfamily Genes | Signaling |
| XM_397248 | GB14664 | Tak1 | IMD | Signaling |
| XM_623562 | GB14317 | IGFn3-8 | IG Superfamily Genes | Signaling |
| XM_623568 | GB18362 | D-PIAS | JakSTAT | Signaling |
| XM_623574 | GB14642 | IGFn3-1 | IG Superfamily Genes | Signaling |
| XM_623948 | GB11373 | Rac | RAC1 protein | Signaling |
| XM_623999 | GB16397 | pelle | Toll/TLR | Signaling |
| XM_624192 | GB14382 | CTL8 | C-lectin domain | Signaling |
| XM_624204 | GB10539 | TRAF2 | Toll/TLR | Signaling |
| XM_624416 | GB18949 | SOCS | JakSTAT | Signaling |
| XM_624969 | GB13677 | cactin | Toll/TLR | Signaling |
| XR_409811 | GB10912 | IGFn3-6 | IG Superfamily Genes | Signaling |
| XR_409812 | GB10912 | IGFn3-6 | IG Superfamily Genes | Signaling |
| XR_409962 | GB13522 | MAPKKK9 | MAPK | Signaling |
| XR_412241 | GB15050 | CTL9 | C-lectin domain | Signaling |
| AY268031 | GB19066 | dorsal-1A | Toll/TLR | Signaling |
| AY268031 | GB19537 | Dorsal-1B | Toll/TLR | Signaling |
| DB756508 | GB13742 | relish | IMD | Signaling |
| DB774085 | GB17879 | PGRP-S3 | PGRP | Signaling |
| EF631982 | GB18949 | SOCS | JakSTAT | Signaling |
| FJ546099 | GB19961 | B-gluc2 | GNBPs | Signaling |
| HP491640 | GB19683 | AmSCR-B10 | Scav. Receptor B | Signaling |
| HP536649 | GB19498 | dUbc13 | IMD | Signaling |
| HP578203 | GB10655 | cact-1 | Toll/TLR | Signaling |
| HQ824709 | GB11358 | IGFn3-2 | IG Superfamily Genes | Signaling |
| NM_001013361 | GB15177 | 18-w | Toll/TLR | Signaling |
| NM_001242997 | GB17330 | CTL5 | C-lectin domain | Signaling |
| XM_001120871 | GB12538 | GRAAL/Tequila-like | Scav. Receptor A | Signaling |
| XM_001120881 | GB16388 | AmSCR-B8 | Scav. Receptor B | Signaling |
| XM_001121036 | GB15371 | PGRP-S1 | PGRP | Signaling |
| XM_001121888 | GB18767 | PPOAct | PPO | Signaling |
| XM_001122420 | GB15640 | PSH LIKE | Toll/TLR | Signaling |
| XM_001122446 | GB14642 | IGFn3-1 | IG Superfamily Genes | Signaling |
| XM_001123170 | GB18732 | PSH LIKE | Toll/TLR | Signaling |
| XM_003250384 | GB20003 | Hemomucin | Cellular response | Signaling |
| XM_003250988 | GB12004 | Jra | JNK | Signaling |
| XM_006557242 | GB16401 | MAPK(basket) | MAPK | Signaling |
| XM_006557421 | GB18049 | CTL3 | C-lectin domain | Signaling |
| XM_006558112 | GB15987 | IGFn3-3 | IG Superfamily Genes | Signaling |
| XM_006558639 | GB14382 | CTL8 | C-lectin domain | Signaling |
| XM_006559982 | GB14962 | Draper | Phagocytosis | Signaling |
| XM_006559982 | GB14962 | laminin-EGF-like | EGF Family | Signaling |
| XM_006561053 | GB14645 | Eater-like | EGF Family | Signaling |
| XM_006561054 | GB12933 | IGFn3-14 | IG Superfamily Genes | Signaling |
| XM_006562122 | GB16060 | IGFn3-9 | IG Superfamily Genes | Signaling |
| XM_006562362 | GB19582 | NEC LIKE | Toll/TLR | Signaling |
| XM_006562720 | GB18520 | Toll (TLR) | Toll/TLR | Signaling |
| XM_006562853 | GB16299 | Toll-10 | Toll/TLR | Signaling |
| XM_006564416 | GB13522 | MAPKKK9 | MAPK | Signaling |
| XM_006564466 | GB19649 | Corin-like | Scav. Receptor A | Signaling |
| XM_006564519 | GB16711 | Hemolectin | Cellular response | Signaling |
| XM_006565050 | GB17106 | IKKgamma-kenny | IMD | Signaling |
| XM_006565164 | GB16397 | pelle | Toll/TLR | Signaling |
| XM_006565440 | GB12605 | TEP7 | TEP | Signaling |
| XM_006565671 | GB15344 | IGFn3-15 | IG Superfamily Genes | Signaling |
| XM_006566758 | GB15645 | NFAT | NFAT | Signaling |
| XM_006566936 | GB10506 | AmSCR-B1 | Scav. Receptor B | Signaling |
| XM_006567219 | GB19916 | AmSCR-B9 | Scav. Receptor B | Signaling |
| XM_006567688 | GB16422 | Hopscotch | JakSTAT | Signaling |
| XM_006567774 | GB19901 | puckered | JNK | Signaling |
| XM_006567803 | GB11846 | IGFn3-7 | IG Superfamily Genes | Signaling |
| XM_006567954 | GB14317 | IGFn3-8 | IG Superfamily Genes | Signaling |
| XM_006569288 | GB11807 | IGFn3-10 | IG Superfamily Genes | Signaling |
| XM_006569295 | GB14314 | AmSCR-B6 | Scav. Receptor B | Signaling |
| XM_006570012 | GB18324 | Galectin-2 | Galectin | Signaling |
| XM_006570671 | GB17018 | Angiopoietin | Fibrinogen | Signaling |
| XM_006571575 | GB14044 | PSH LIKE | Toll/TLR | Signaling |
| XM_006571997 | GB17012 | NEC LIKE | Toll/TLR | Signaling |
| XM_392090 | GB13360 | lox2-like | Scav. Receptor A | Signaling |
| XM_392379 | GB10026 | Galectin-1 | Galectin | Signaling |
| XM_392617 | GB10469 | IGFn3-13 | IG Superfamily Genes | Signaling |
| XM_393712 | GB17781 | Toll-6 | Toll/TLR | Signaling |
| XM_394485 | GB13520 | cact-2 | Toll/TLR | Signaling |
| XM_394528 | GB11320 | RIP1 | MAPK | Signaling |
| XM_395355 | GB13808 | CTL12 | C-lectin domain | Signaling |
| XM_395773 | GB11810 | CTL10 | C-lectin domain | Signaling |
| XM_396085 | GB13813 | AmSCR-B5 | Scav. Receptor B | Signaling |
| XM_397181 | GB18923 | STAT92E | JakSTAT | Signaling |
| XM_397416 | GB18789 | TEPA | TEP | Signaling |
| XM_397430 | GB15549 | AmSCR-B7 | Scav. Receptor B | Signaling |
| XM_623132 | GB15273 | IKK-ird5 | IMD | Signaling |
| XM_624291 | GB19013 | CTL11 | C-lectin domain | Signaling |
| XM_624414 | GB17961 | Tollip | Toll/TLR | Signaling |
| XM_624533 | GB14975 | CTL7 | C-lectin domain | Signaling |
| XM_625153 | GB11883 | cact-3 | Toll/TLR | Signaling |
| XR_409148 | GB19452 | B-gluc1 | GNBPs | Signaling |

**Supplementary figure S2**. Comparison of transcript abundance (adjusted read counts) in *Apis mellifera* worker bees. Expression in nurses versus foragers is shown in a) the hypopharyngeal gland (HPG gland), b) mandibular gland, c) midgut, and d) Malpighian tubules. Points indicate transcripts mapped to particular genes, and colored points indicate differentially expressed genes detected using edgeR. Red points represent genes upregulated in nurses and blue points those upregulated in forager worker bees. Outlined points are focal genes with putative antimicrobial, immune signaling, and detoxification functions.

**Supplementary figure S3** Comparison of genes differentially expressed between nurses and foragers of honeybee *Apis mellifera* from tissue-specific transcriptomic analysis (RNA-seq data) using edgeR and DEseq2. Genes were considered differentially expressed if the BH-adjusted P-value <0.05.

**Supplementary table S4.** Gene-specific primers used in qPCR analysis. Primers spanning exon junctions were designed with NCBI’s primer blast tool (primer 3 plus the most recent build of the honey bee genome, 4.5).

| **Gene** | **genbank ID** | **forward primer** | **reverse primer** |
| --- | --- | --- | --- |
| apisimin | NM_001011582.1 | GTCGTCCTAGCTGCCTTCTG | GGAAACGACATCCACGTTCG |
| defensin_1 | NM_001011616.2 | GGCTGCACCTGTTGAGGAT | TGTCCTTTGAATGAGAGAAGGTCA |
| hymenoptaecin | NM_001011615.1 | ACAATGGATTATATCCCGACTCGT | CAATGTCCAAGGATGGACGAC |
| cytochrome_P450_9Q3 | XM_006562300.1 | GTAGGGAGAAGTTGGGCACC | GCTACGTTCTCGACGGTCAT |
| cytochrome_P450_6AS4 | XM_395671.4 | TCGTTTTGCGCGATCTTGAC | AAAAAGGTTCGGCTCGTTCG |
| cytochrome_P450_6BD1 | XM_006564436.1 | TGGCGGATTACAAGATACCAGG | AACGGAGAGGATCTGGATGA |
| UDP-glycosyltransferase | XM_006560047.1 | AAGTGTGCTCGCTCATCCAA | GGTATACCGAGAACTGGTACTTCA |
| eIF3-S8 | XM_006564593.1 | AAGAGAGTTGTACGATCAACCA | TCTTCAAAACTGGACAACATGC |
